# *KRT5* missense variant in a Cardigan Welsh Corgi with epidermolysis bullosa simplex

**DOI:** 10.1111/age.13257

**Published:** 2022-08-25

**Authors:** Sarah Kiener, Elizabeth A. Mauldin, Vidhya Jagannathan, Margret L. Casal, Tosso Leeb

**Affiliations:** ^1^ Vetsuisse Faculty, Institute of Genetics University of Bern Bern Switzerland; ^2^ DermFocus University of Bern Bern Switzerland; ^3^ School of Veterinary Medicine University of Pennsylvania Philadelphia Pennsylvania USA

**Keywords:** animal model, *Canis lupus familiaris*, dermatology, dog, genodermatosis, precision medicine, skin, veterinary medicine

## Abstract

Epidermolysis bullosa (EB) is a group of blistering disorders that includes several subtypes, classified according to their level of cleavage. Typical clinical signs are blisters and erosions resulting from minimal trauma. The disease has been described in many mammalian species and pathogenic variants in at least 18 different genes have been identified. In the present study, we investigated a Cardigan Welsh Corgi with congenital clinical signs consistent with epidermolysis bullosa. The puppy had blisters and erosions on the paw pads, and the oral mucosa. Histologic examination demonstrated the typical clefting between the dermis and epidermis and confirmed the clinical suspicion. We obtained whole genome sequencing data from the affected puppy and searched for variants in candidate genes known to cause EB. This revealed a heterozygous missense variant, *KRT5*:p.(E476K), affecting the highly conserved KLLEGE motif of keratin 5. The mutant allele in the affected puppy arose owing to a de novo mutation event as it was absent from both unaffected parents. Knowledge of the functional impact of *KRT5* variants in other species together with the demonstration of the de novo mutation event establishes *KRT5*:p.(E476K) as causative variant for the observed EBS.

## INTRODUCTION

Epidermolysis bullosa (EB) comprises a heterogeneous group of blistering disorders. They are characterized by blisters and erosions resulting from minimal trauma (Fine et al., 2008; Has et al., 2018). Classification into different types of EB is based on the level of cleavage. These are EB simplex (EBS), with a level of cleavage in the basal layer of the epidermis, junctional EB, with a level of cleavage at the dermo‐epidermal junction, and dystrophic EB, with a level of cleavage just below the basal membrane. The fourth category, Kindler EB, with a mixed level of cleavage, is a rare EB type with a very severe clinical phenotype (Has et al., 2020). The human classification is also the basis for the classification of EB types in animals (Medeiros & Riet‐Correa, 2015).

Hereditary forms of EB follow a monogenic mode of inheritance with autosomal dominant or autosomal recessive inheritance. So far, a large number of disease causing alleles have been identified in at least 18 genes coding for structural proteins in human skin (Fine et al., 2008; Has et al., 2014; Lemke et al., 2014).

In domestic animals, cases of EB have been described but molecular studies are rare. Causative genetic variants have been identified in dogs, cats, horses, cattle and sheep (Medeiros & Riet‐Correa, 2015). In dogs, variants in four different genes, namely *COL7A1*, *LAMA3*, *LAMB3* and *PLEC*, have been described (Baldeschi et al., 2003; Capt et al., 2005; Garcia et al., 2020; Herrmann et al., 2021; Kiener et al., 2020; Mauldin et al., 2017; Niskanen et al., 2017).

The identification of a pathogenic variant and thus the molecular characterization of a suspected inherited disease is of great value as it provides a fast and minimally invasive definitive diagnosis. Further, it offers the opportunity to give breeding recommendations and may even provide guidance for targeted therapy (Leeb et al., 2022). Therefore, the aim of this study was to characterize the clinical and histopathological phenotype of the affected puppy and to identify the underlying genetic variant using a whole genome sequencing approach.

**FIGURE 1:**
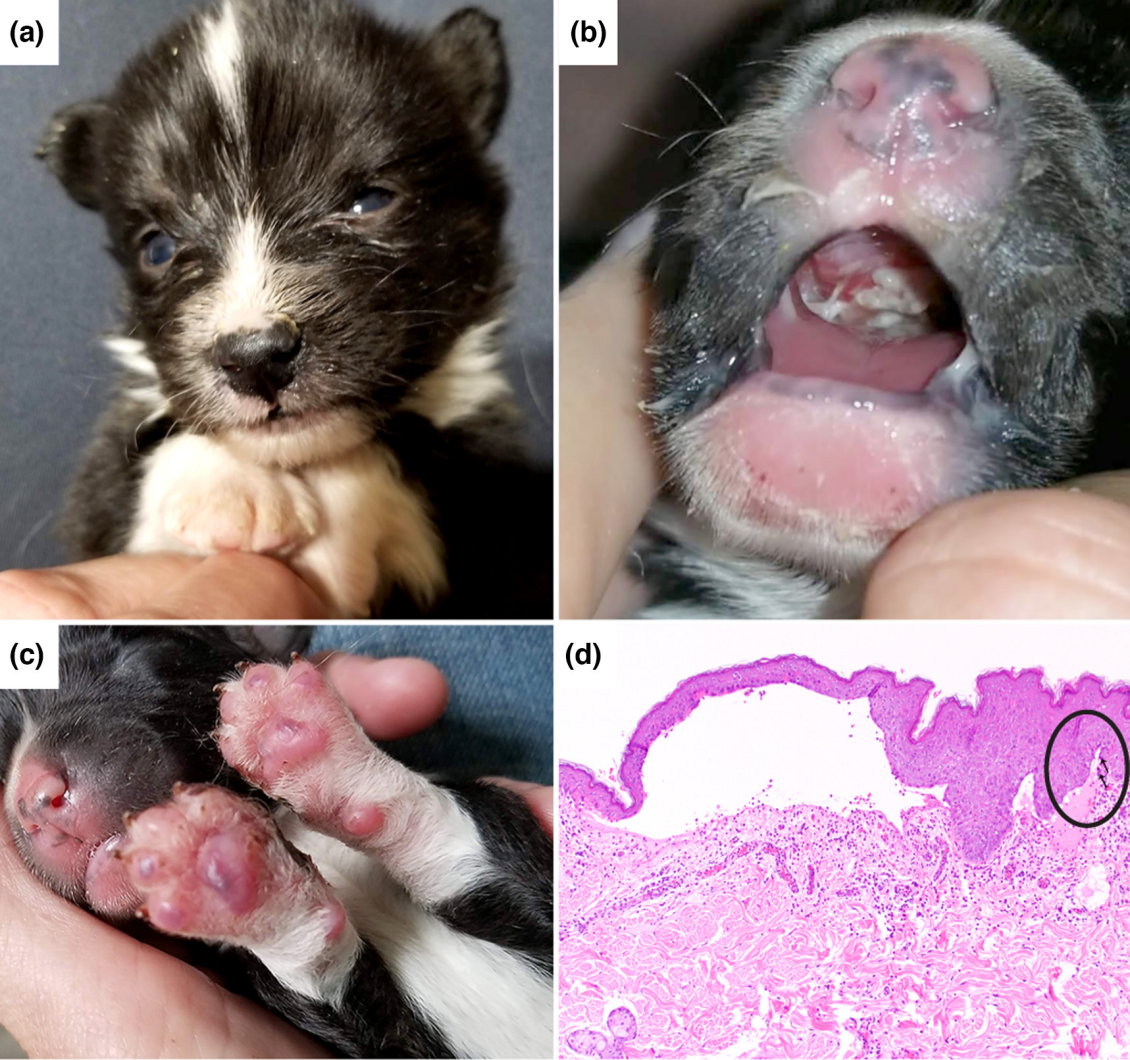
Phenotype of the Cardigan Welsh Corgi affected with epidermolysis bullosa. (a) Underweight 12‐day‐old puppy. (b) Extensive ulcers in the oral cavity. Note that the epidermis sloughs with gentle pressure on the perioral skin. (c) Numerous vesicles on the paw pads. (d) Broad subepidermal vesicle with remnants of basal keratinocytes at the marginal base of the blister (thin arrows in black circle). H&E 10×.

We examined a Cardigan Welsh Corgi puppy with skin blistering and erosions that were noticed shortly after birth. It was one of a litter of three puppies, in which both other littermates and the parents were healthy. The affected dog was smaller than the littermates. Vesicles and ulcers were extensively present in the oral cavity, lips and paw pads (Figure 1). Gentle pressure on the skin near affected areas would result in sloughing of the adjacent epidermis (positive Nikolsky sign; Maity et al., 2020). The puppy was given supplementary nutrition and adopted by a veterinarian at 10 weeks of age. At that time, the lesions in the oral cavity had healed and the dog was able to eat normally. The dog continued to have waxing and waning lesions on the paw pads, ears, axilla and groin. Blisters would arise intermittently, ulcerate and heal with scarring. At 1 year of age, four skin punch biopsies from lesional areas were obtained under general anesthesia. Histopathologic examination revealed intact subepidermal vesicles along with ulcers, granulation tissue and regions of dermal scarring. Small remnants of basal keratinocytes were evident at the margins of the vesicles and scattered basal keratinocytes were apoptotic. The dog was still alive at 17 months of age (at the time of writing this paper).

**FIGURE 2:**
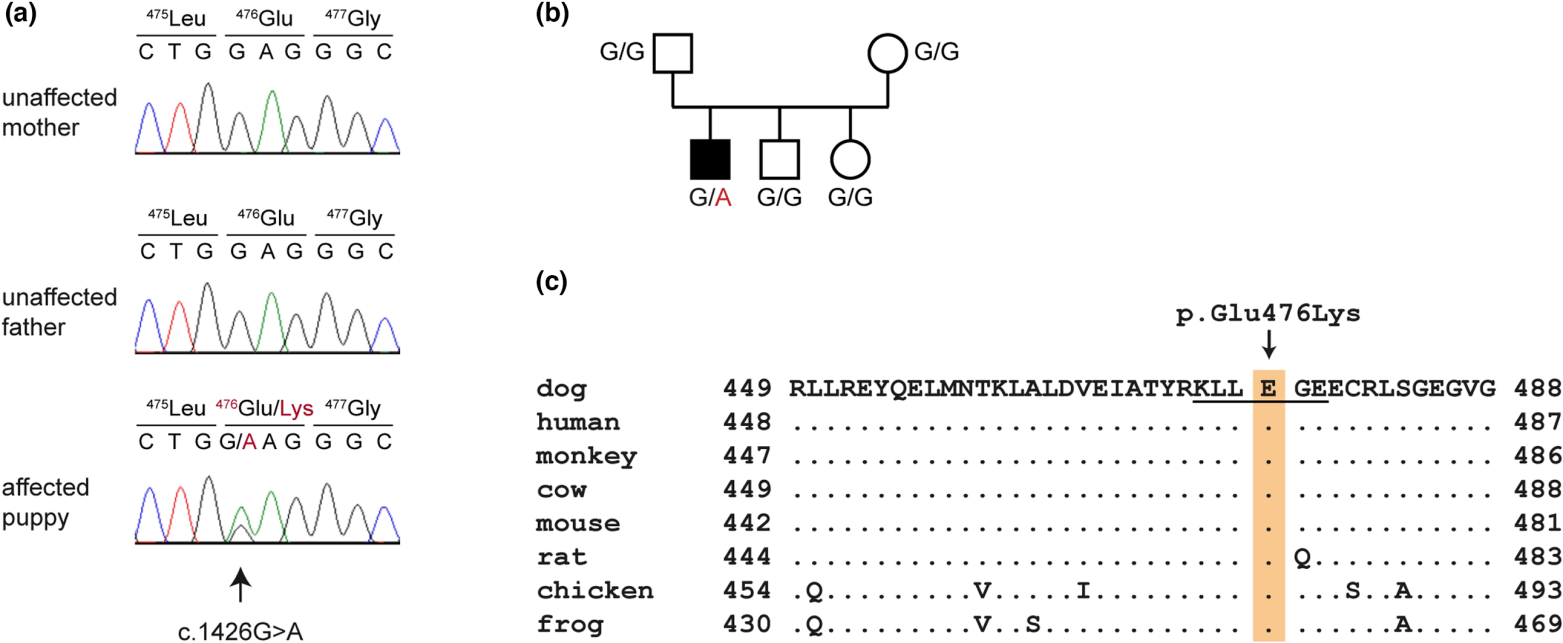
Details of the identified variant in *KRT5*. (a) Electropherograms showing the *KRT5*:C.1426G>a missense variant. The variable position is indicated with an arrow and the amino acid translations are shown. (b) Pedigree of the family of the affected dog. Squares represent males and circles represent females. The filled symbol indicates the affected dog. The genotypes for each animal at the position of the identified *KRT5* variant are given. (c) Multiple‐species alignment of the KRT5 protein in the region of the p.E476K variant. The variant affects the first glutamate residue of the highly conserved KLLEGE motif (underlined).

Based on the characteristic clinical signs and the early age of onset, we suspected an underlying genetic defect and performed a genetic analysis. We therefore collected EDTA blood samples of the complete family and isolated genomic DNA from peripheral leukocytes. The genome of the affected puppy was sequenced at 22× coverage on an Illumina Novaseq 6000 instrument. Mapping and variant calling with respect to the UU_Cfam_GSD_1.0 reference genome assembly were performed as described (Jagannathan et al., 2019). We searched for private variants by comparing the sequencing data of the affected puppy with 563 control genomes and identified 134 heterozygous and six homozygous private protein‐changing variants (Tables S1 and S2). These included a heterozygous single nucleotide substitution in *KRT5*, Chr27:44080887C>T (UU_Cfam_GSD_1.0), which is a well‐characterized candidate gene for EB. This missense variant, NM_001346035.1:c.1426G>A, is predicted to change a conserved glutamate in keratin 5, NP_001332964.1:p.(E476K). Sanger sequencing confirmed the presence of the variant in the affected dog and showed that it had arisen de novo, as both parents were homozygous for the wild‐type allele (Figure 2).

*KRT5* encodes keratin 5, which is a type II keratin and expressed in the basal keratinocytes. Together with keratin 14 it builds intermediate filaments which are important for cell structure and stability (Arin, 2009). Both keratins comprise two helical rod domains, which assemble into alpha‐helical coiled‐coil heterodimers (Fuchs & Cleveland, 1998; Stephens et al., 1997). When this structural framework is disorganized, cells become fragile and tend to rupture under stress (Fuchs & Cleveland, 1998).

The identified heterozygous missense variant E476K affects the first glutamate residue of the KLLEGE motif at the end of the rod domain of keratin 5. This motif is highly conserved and important for filament assembly and stability (Letai et al., 1992, 1993; Müller et al., 2006; Wilson et al., 1992). Previously, variants affecting the first and second glutamate residues of the KLLEGE motif of keratin 5 have been described in humans and cattle with EBS (Ford et al., 2005; García et al., 2011; Lane et al., 1992; Schuilenga‐Hut et al., 2003; Stephens et al., 1997; Yasukawa et al., 2006). The human variant E475K, homologous to the canine E476K variant, was reported to cause EBS in three human patients (Schuilenga‐Hut et al., 2003; Yasukawa et al., 2006).

Human patients with a E477K variant affecting the second glutamate in the KLLEGE motif may develop a particularly severe and sometimes lethal EBS phenotype. Molecular modeling demonstrated the transition from a negatively charged glutamate to a positively charged lysine on the surface of the 2B domain of keratin 5 that is in direct contact with the 2B domain of keratin 14 (Lalor et al., 2019).

Monoallelic *KRT5* variants leading to the production of an aberrant keratin 5 protein are a common cause for EBS (Has et al., 2020). They act in a dominant negative manner, in which the abnormal protein produced by the mutant allele interferes with the normal protein in the process of keratin filament assembly (Yasukawa et al., 2006). Knowledge of the functional impact of *KRT5* missense variants at this position in humans and cattle, together with the demonstrated de novo mutation event and the absence of the mutant allele from a large number of control dogs, establishes the pathogenicity of the detected heterozygous *KRT5*:p.(E476K) variant according to human standards (Richards et al., 2015). The molecular analysis refined the diagnosis in the affected dog from EB to EBS. To the best of our knowledge, this is the first report of a *KRT5* variant causing EBS in dogs.

## CONFLICT OF INTEREST

The author declare no conflicts of interest.

## DATA AVAILABILITY STATEMENT

Accession numbers for the whole genome sequence data sequences are given in Table S1.

## Supporting information


Table S1

Table S2
